# Relationships Between Element Contents in Polish Children’s and Adolescents’ Hair

**DOI:** 10.1007/s12011-017-0987-1

**Published:** 2017-03-10

**Authors:** Maria Długaszek, Wojciech Skrzeczanowski

**Affiliations:** Institute of Optoelectronics, Military University of Technology, gen. Sylvester Kaliski 2, 00-908 Warsaw 49, Poland

**Keywords:** Children and adolescents, Gender, Age, Hair, Essential and toxic elements

## Abstract

Environment, sex, and age are the main factors which determine the elemental composition of hair. The objective of the study is to determine the contents of calcium (Ca), magnesium (Mg), zinc (Zn), copper (Cu), iron (Fe), lead (Pb), and cadmium (Cd) in girls’ and boys’ hair in five age groups (within 1–19-year range) corresponding to successive human ontogenesis phases as well as to evaluate the relationships between these elements. Quantitative analysis has been carried out using atomic absorption spectrometry (AAS). Experimental results were analyzed using classic and principal component (PCA) statistical analyses. In particular, differences between contents of particularly Ca, Mg, and Zn in girls’ and boys’ hair were found, and substantial differences between age groups were stated. In general, larger amounts of Ca, Mg, and Zn as compared to boys’ hair have been observed for girls’ hair and higher toxic element (Pb, Cd) contents for boys were measured in some age groups. An increasing trend was found for bioelements (Ca, Mg, Zn) both for girls and boys in all age groups, while for Cu and Fe content, changes are insignificant and even decreasing for teenagers. The most frequently correlating element pairs are Ca–Mg, Ca–Zn, Mg–Zn, and Pb–Cd. Classic and PCA statistics show, in general, a satisfactory consistence. The elemental composition of hair varies depending on the gender and age of children and young people.

## Introduction

Recently, a huge interest in applications of hair analysis in medical diagnostics has been observed. Publications describing composition of various elements in hair of healthy and sick people exposed to excessive amounts of these elements or threatened by deficiency of elements essential for life have been published [1–11]. Elemental analysis of hair has both advantages and disadvantages. Many factors determine element content in hair, for instance individual characteristics such as sex, age, functioning of endocrine glands, health condition, effectiveness of absorption and excretion processes, diet, and also natural and occupational environment. On the other hand, the amount of elements in hair is more stable than elemental content in blood and urine and reflects mineral metabolism of the human body over a period of few months [1–4]. The chelating capabilities of hair result, first of all, from the presence of sulfur amino acids being a part of keratin—a major protein component of hair structure. Metal complexing properties are also observed for melanin—a pigment coloring hair [2, 3]. Changes in children’s and adolescents’ organisms are very dynamic. This also applies to hair. Children’s hair differs from that of adults. Its number, density, thickness, and color change with age [2, 3]. Other factors, such as absorption and excretion and the level of some hormones, influence the mineral composition of hair [1–4]. In some papers [1, 2, 5–11], differences in element composition in women’s and men’s hair and correlations between element concentrations and age of tested people were observed. Significant variability was observed for children and adolescents [7]. Children and young people are particularly susceptible to adverse environmental conditions and poor nutrition; therefore, we should pay special attention to fast, safe, and relevant methods of assessing their health. Elemental analysis of hair can be particularly useful in case of children in the monitoring of environmental exposure and assessment of nutrition status, but it is necessary to establish appropriate reference ranges for different elements depending, mainly, on the age and sex of the child. Still, hair has been rarely examined in reference to sex and successive age phases of young people and authors [1, 8–10] underline the need for continuation of research on this subject.

Physiological processes maintaining homeostasis depend among others on the composition and concentration of elements as well as their proportions in the body. The excess or deficiency of some of them influences the content of other elements, for example, Ca, Mg, Zn, and toxic metals Pb and Cd. In the paper, an attempt was made to evaluate the concentrations of essential elements such as Ca, Mg, Zn, Cu, and Fe and toxic metals, i.e., Pb and Cd in children’s and adolescents’ hair (girls and boys) in age groups corresponding to successive phases of human ontogenesis (i.e., toddlerhood, play age, middle childhood, and adolescence) as well as to find the mutual quantitative correlations between the elements.

## Materials and Methods

### Study Population

**Fig. 1:**
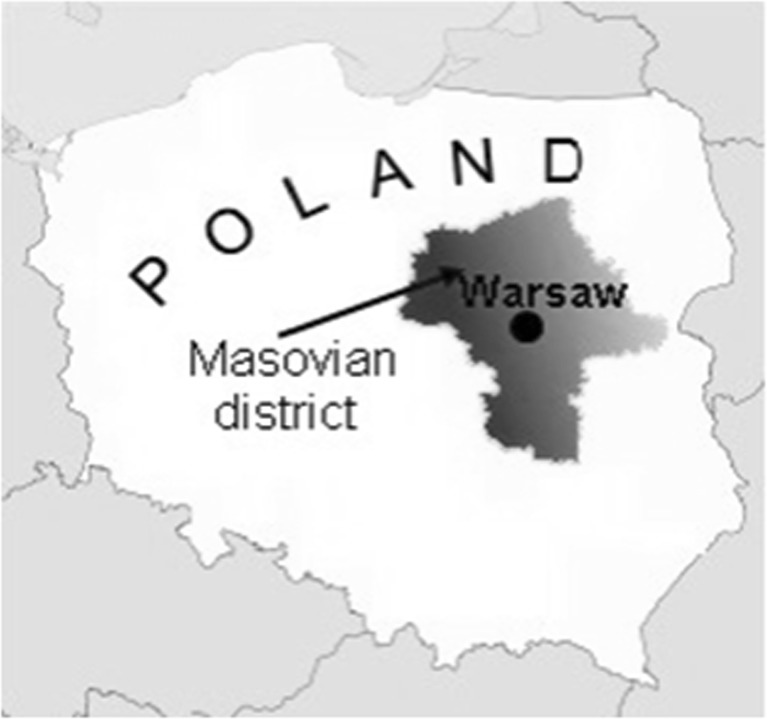
Geographical location of sampling area

Samples of healthy children’s and adolescents’ hair were taken from 235 girls and 353 boys coming from Warsaw and Mazovian District. The location of the sampling area (obtained from GPS) is shown in Fig. 1. Hair was collected from 2007 to 2012. Young people were divided into five age groups: 1–3-year-old toddlers, 4–6-year-old preschoolers, 7–10–year-old and 11–15-year-old schoolgirls and schoolboys, and 16–19-year-old adolescents (Table 1). Parents of all young participants who took part in our research expressed their consent to carry out the tests.

**Table 1:** Percentile (*P*) values of content of elements in girls’ and boys’ hair according to age group (μg/g)

Girls		Ca	Mg	Zn	Cu	Fe	Pb	Cd	Boys		Ca	Mg	Zn	Cu	Fe	Pb	Cd
1–3	P10	83	4	42	7	8	0.3	0.05	1–3	P10	64	4	59	7	7	0.3	0.05
(*n* = 70)	P25	119	6	67	8	9	0.6	0.05	(*n* = 82)	P25	88	5	71	8	8	0.5	0.05
	Median	176	8	101	10	11	0.9	0.05		Median	123*	6	95	9	10	0.9	0.05
	P75	283	12	155	11	13	1.3	0.05		P75	187	8	126	11	13	1.6	0.05
	P90	355	18	204	15	15	1.6	0.06		P90	253	13	167	14	16	2.7	0.11
4–6	P10	66	4	57	8	8	0.3	0.05	4–6	P10	67	4	54	7	7	0.3	0.05
(*n* = 62)	P25	112	6	80	9	9	0.4	0.05	(*n* = 127)	P25	99	6	69	8	8	0.5	0.05
	Median	178	10	118	11	10	0.7	0.05		Median	147	8	105	9	10	0.8	0.05
	P75	302	15	141	14	13	1.1	0.05		P75	231	12	139	11	12	1.4	0.05
	P90	380	22	170	23	15	1.5	0.05		P90	321	17	181	15	16	2.2	0.08
7–10	P10	101	5	79	7	6	0.2	0.05	7–10	P10	98	4	75	8	6	0.3	0.05
(*n* = 50)	P25	176	8	114	9	7	0.4	0.05	(*n* = 75)	P25	150	8	109	8	7	0.6	0.05
	Median	252	14	163	11	9	0.7	0.05		Median	186*	11*	138**	10	9	1.0*	0.05
	P75	535	24	189	16	11	1.0	0.05		P75	313	13	165	13	11	1.5	0.06
	P90	733	41	238	33	15	2.3	0.06		P90	456	27	181	20	13	2.8	0.09
11–15	P10	258	16	159	8	5	0.2	0.05	11–15	P10	153	8	126	7	5	0.2	0.05
(*n* = 31)	P25	374	18	163	9	6	0.3	0.05	(*n* = 53)	P25	186	10	151	8	6	0.4	0.05
	Median	556	24	189	12	6	0.4	0.05		Median	250***	15***	169*	9*	7	0.8	0.05
	P75	1050	36	216	14	8	0.8	0.05		P75	407	27	191	11	8	1.1	0.05
	P90	1288	40	236	17	9	1.2	0.05		P90	592	36	232	17	11	2.0	0.05
16–19	P10	356	18	152	5	6	0.2	0.05	16–19	P10	213	12	139	7	6	0.1	0.05
(*n* = 25)	P25	448	22	176	10	6	0.2	0.05	(*n* = 17)	P25	241	17	168	9	6	0.3	0.05
	Median	730	27	204	12	8	0.4	0.05		Median	325**	21	179	11	7	0.3	0.05
	P75	1269	33	271	13	11	0.5	0.05		P75	516	30	202	13	8	0.9	0.05
	P90	1592	51	397	22	28	0.7	0.05		P90	1139	86	229	17	9	1.4	0.06

Statistically significant differences between elements’ amount in girls’ and boys’ hair (*p < 0.05, **p < 0.01, ***p < 0.001, Kolmogorov-Smirnov test)

### Analytical Procedures

Hair samples (untreated chemically) were taken from several points of head in amount of about 0.3 g; 3–4-cm-long fragments counting from skin were used for elemental analysis. The samples of hair were washed (with aqueous solution of detergent free from metal ions, redistilled and deionized water (0.06 μS/cm), and acetone in a Soxhlet apparatus for 25 min) and next dried in a laboratory drier (60 °C) and in a desiccator. The samples of hair were mineralized in the mineralization system, in a mixture (3:1, *v*/*v*) of acids HNO_3_ and HClO_4_ (Cheman S.A.) at boiling temperature, during about 20–30 min. Concentrations of Ca, Mg, Zn, Cu, and Fe were determined by flame atomic absorption spectrometry (FAAS), and amounts of Pb and Cd were measured by graphite furnace atomic absorption spectrometry (GFAAS) with the use of atomic absorption spectrometer AVANTA Σ (GBC Scientific, Dandenong, Australia). Measurements of Ca and Mg concentrations were performed in the presence of LaCl_3_ (Fisher Scientific) and Pb and Cd in the presence of NH_4_H_2_PO_4_ (Merck) and NH_4_NO_3_ (Aldrich) as modifiers. All used reagents were spectrally pure, and every stage during the processes of sample preparation and instrumental analysis was carried out under dust-free conditions corresponding to the requirements of the AAS method.

The accuracy of analytical procedures was verified based on the reference material (human hair NCS ZC 81002) analysis. The values ranged from 93.4% for Pb to 109.1% for Mg. Precision of instrumental procedures fluctuated from 4.3% (Zn) to 8.7% (Ca). Detailed information on instrumental analysis and the results of validation procedures have already been presented in previous publications [5, 6, 11].

### Statistical Analyses

The results obtained in this study were elaborated using the statistical analysis software Statistica versions 9 and 10 (StatSoft, Cracow, Poland). Distributions of variables were non-parametric (Shapiro-Wilk test), so adequate statistics were used for interpreting the data (Kolmogorov-Smirnov test, Kruskal-Wallis test, and the Spearman’s rank test). Moreover, the PCA was used in multivariable comparative analysis. The PCA allows to compare objects or sets consisting of many variables and quickly find the most important properties of the data. Advanced statistical methods using graphical presentation are more and more frequently used in the processing of data obtained in the study of biological samples, such as biological aerosols [12]. Due to complex mechanisms related to final elemental content in human hair, it was reasonable to assume that multivariable statistical analysis such as PCA, which includes many input variables, can be an additional effective analytical tool for experimental data [13, 14].

## Results

**Fig. 2:**
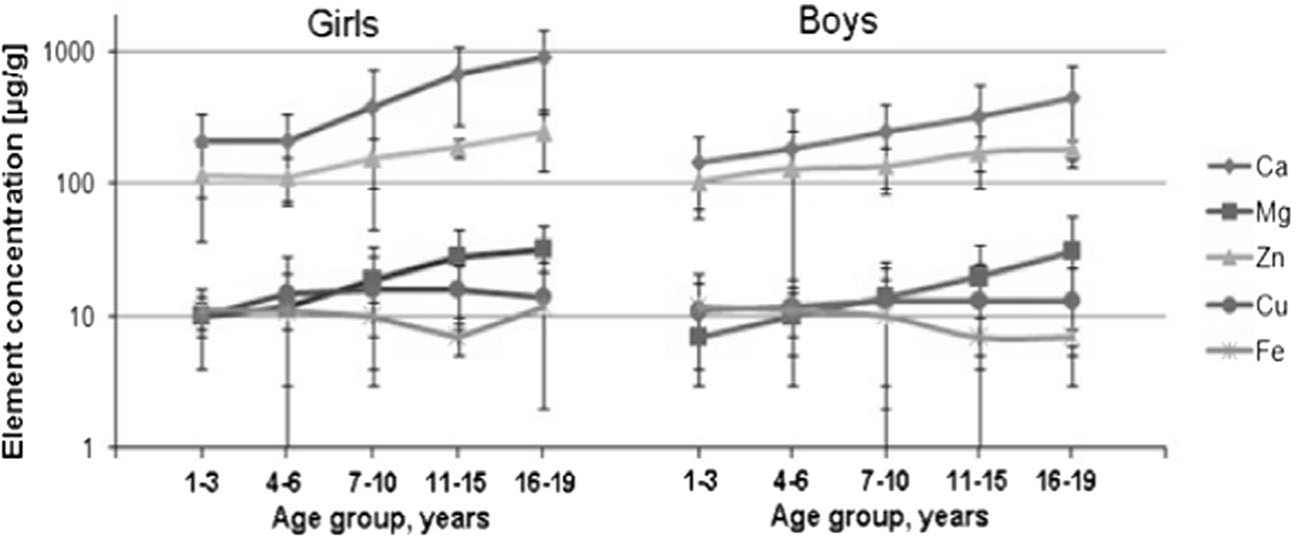
The mean content (μg/g) of elements in children’s and adolescents’ hair according to age groups (the significance of differences is shown in Tables 1 and 2)

The content of Ca, Mg, Zn, Cu, Fe, Pb, and Cd expressed as 10th, 25th, 50th (median), 75th, and 90th percentiles in children’s and adolescents’ hair is shown in Table 1, while the average and SD values are presented in Fig. 2. In the individual age groups, the differences related to elemental content in hair of tested children and adolescents are clearly seen. In girls’ hair, like in women’s hair [7], larger amounts of Ca, Mg, Zn, and Cu as compared to boys’ hair have been found. However, the largest amounts of toxic metals were found in boys’ hair rather than in girls’ taking into account the range and average values. Largest quantities of Pb were found in the hair of 7–10-year-old boys. Mean values for Pb (0.5–1.6 μg/g) in all boy age groups are higher than in girl ones (0.4–1.0 μg/g), and mean values for Cd are also higher in boys hair (0.05–0.07 μg/g in all tested age groups), except for the last one (16–19 years old), in comparison to girls (0.05–0.06 μg/g). In both girls’ and boys’ hair, the amount of Ca, Mg, and Zn demonstrates an upward trend in tested age groups. The Cu concentrations change insignificantly. The lowest Fe content was found in girl’s hair from the 11–15-year-old group. The Fe content decreases with age, but a bit higher values were observed for the 16–19-year-old girl group. The spread of results is comparable for Ca, Mg, Zn, and Pb and is lower for Cu, Fe, and Cd.

In order to establish whether there are statistically significant differences between the amounts of elements in individual age groups, the Kruskal-Wallis test was applied (statistical significance for *p* < 0.05). Results of the analysis point to major significant differences in Ca, Mg, Zn, and Fe contents between individual age groups, both for girls’ and boys’ hair (Table 2). To a lesser extent, these differences apply to Pb content and do not relate to Cu and Cd. Elemental content of children’s hair from the first (1–3 years old), second (4–6 years old), fourth (11–15 years old), and fifth (16–19 years old) groups is similar, and there are no differences between girls’ and boys’ hair substantially from the statistical point of view. The highest variability is observed for the hair of children in the 7–10-year-old group.

**Table 2:** Age groups for which element contents in hair significantly differ from each other

	1–3 (1)		4–6 (2)		7–10 (3)		11–15 (4)		16–19 (5)	
	G^a^	B^b^	G	B	G	B	G	B	G	B
Ca	3, 4, 5	3, 4, 5	3, 4, 5	3, 4, 5	1, 2, 4, 5	1, 2, 5	1, 2, 3	1, 2	1, 2, 3	1, 2, 3
Mg	3, 4, 5	3, 4, 5	4, 5	4, 5	1, 4, 5	1, 4, 5	1, 2, 3	1, 2, 3	1, 2, 3	1, 2, 3
Zn	3, 4, 5	3, 4, 5	3, 4, 5	3, 4, 5	1, 2, 4, 5	1, 2, 4, 5	1, 2, 3	1, 2, 3	1, 2, 3	1, 2, 3
Fe	4, 4	3, 4, 5	4	4, 5	4	1, 4	1, 2, 3	1, 2, 3	1	1, 2
Pb	4, 5	5	5	5	5	5	1		1, 2, 3	1, 2, 3

^a^Girls

^b^Boys

On the basis of the Spearman test, we found positive and statistically significant correlations in both girls’ and boys’ hair for Ca, Mg, and Zn, while negative—for Fe and Pb and age of the children. Only in case of Cu and Cd in the hair of boys, there are no significant correlations between their content and age of the children (Table 3).

**Table 3:** Spearman coefficient values for correlation between content of elements in hair and age of all girls and boys

	Ca	Mg	Zn	Cu	Fe	Pb	Cd
Girls	0.57*	0.60*	0.56*	0.17*	−0.39*	−0.33*	−0.17*
Boys	0.48*	0.51*	0.49*	0.05	−0.37*	−0.12*	−0.01

*Statistically significant differences between elements’ amount in girls’ and boys’ hair (*p* < 0.05)

The analysis of correlations (the Spearman test) revealed a largest number of correlating elements in statistically significant way for the 7–10-year-old girl group and comparable number for the 4–6-, 7–10-, and 11–15-year-old boy groups (Table 4). In all age groups, Ca and Mg correlate both for girls and boys; often, correlations between Ca and Zn, Mg and Zn, and Pb and Cd are observed. Negative correlations were found for Ca and Pb, for Mg and Pb, and for Zn and Cd.

**Table 4:** Statistically significant correlations between contents of elements in girls’ and boys’ hair in successive age groups (*p* < 0.05) and corresponding values of Spearman correlation coefficients

1–3		4–6		7–10		11–15		16–19	
Girls									
Ca–Mg	0.77	Ca–Mg	0.81	Ca–Mg	0.83	Ca–Mg	0.69	Ca–Mg	0.40
Ca–Zn	0.37	Ca–Zn	0.38	Ca–Zn	0.59	Ca–Zn	0.39		
Mg–Zn	0.32	Mg–Zn	0.43	Ca–Cu	0.47	Ca–Fe	0.40		
Zn–Fe	−0.29	Mg–Cu	0.34	Ca–Pb	0.31				
Pb–Cd	0.35	Pb–Cd	0.34	Mg–Zn	0.62				
				Mg–Cu	0.47				
				Mg–Fe	−0.37				
				Mg–Cd	−0.29				
				Zn–Fe	−0.54				
				Cu–Pb	0.36				
				Fe–Cd	0.34				
				Pb–Cd	0.34				
Boys									
Ca–Mg	0.78	Ca–Mg	0.76	Ca–Mg	0.63	Ca–Mg	0.76	Ca–Mg	0.78
Ca–Cu	0.35	Ca–Zn	0.23	Ca–Zn	0.58	Ca–Zn	0.39	Mg–Fe	−0.72
Mg–Zn	0.26	Ca–Fe	−0.18	Ca–Fe	−0.25	Mg–Zn	0.27	Zn–Fe	0.59
Fe–Pb	0.38	Mg–Zn	0.34	Mg–Zn	0.56	Mg–Pb	−0.28	Cu–Fe	−0.53
Fe–Zn	−0.37	Zn–Fe	−0.32	Zn–Fe	−0.31	Zn–Fe	−0.31		
Fe–Cd	0.30	Zn–Pb	−0.23	Zn–Pb	−0.23	Zn–Pb	−0.38		
Pb–Cd	0.45	Cu–Fe	0.25	Cu–Pb	0.27	Zn–Cd	−0.30		
		Cu–Pb	0.26	Fe–Pb	0.37	Cu–Fe	0.27		
		Fe–Pb	0.48	Fe–Cd	0.43	Fe–Pb	0.36		
		Fe–Cd	0.30	Pb–Cd	0.50	Pb–Cd	0.39		
		Pb–Cd	0.43						

**Fig. 3:**
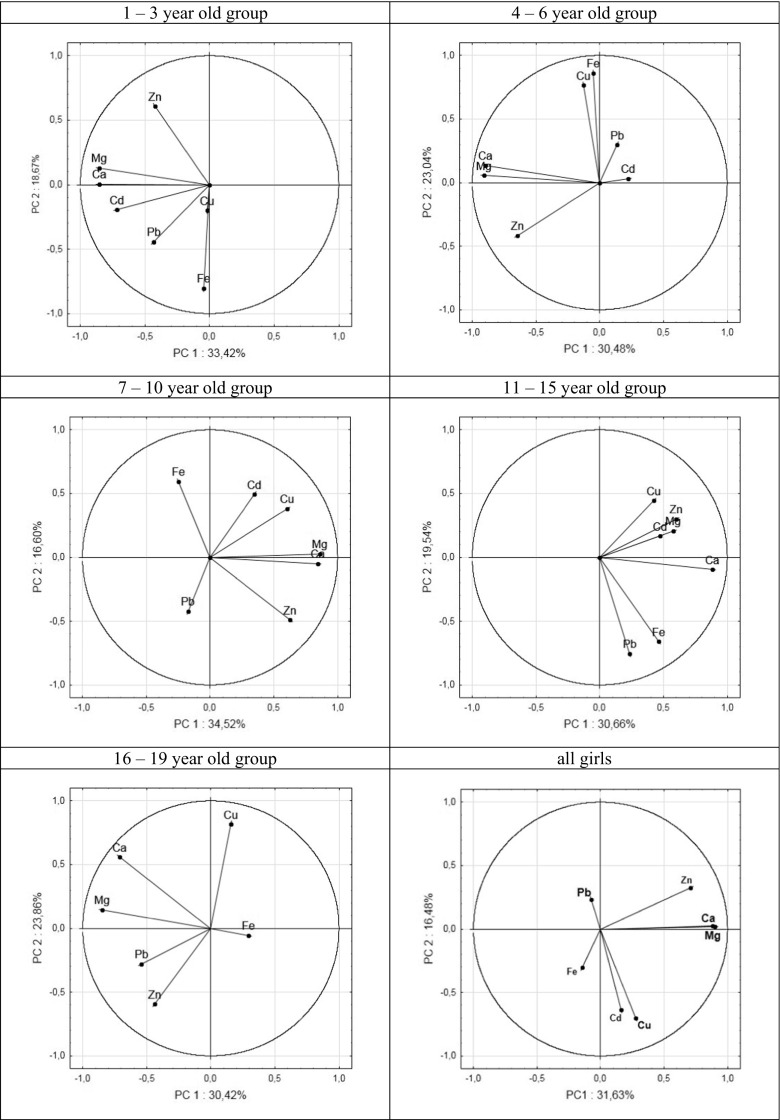
Graphical PCA presentation of relationships between contents of elements in girls’ hair

**Fig. 4:**
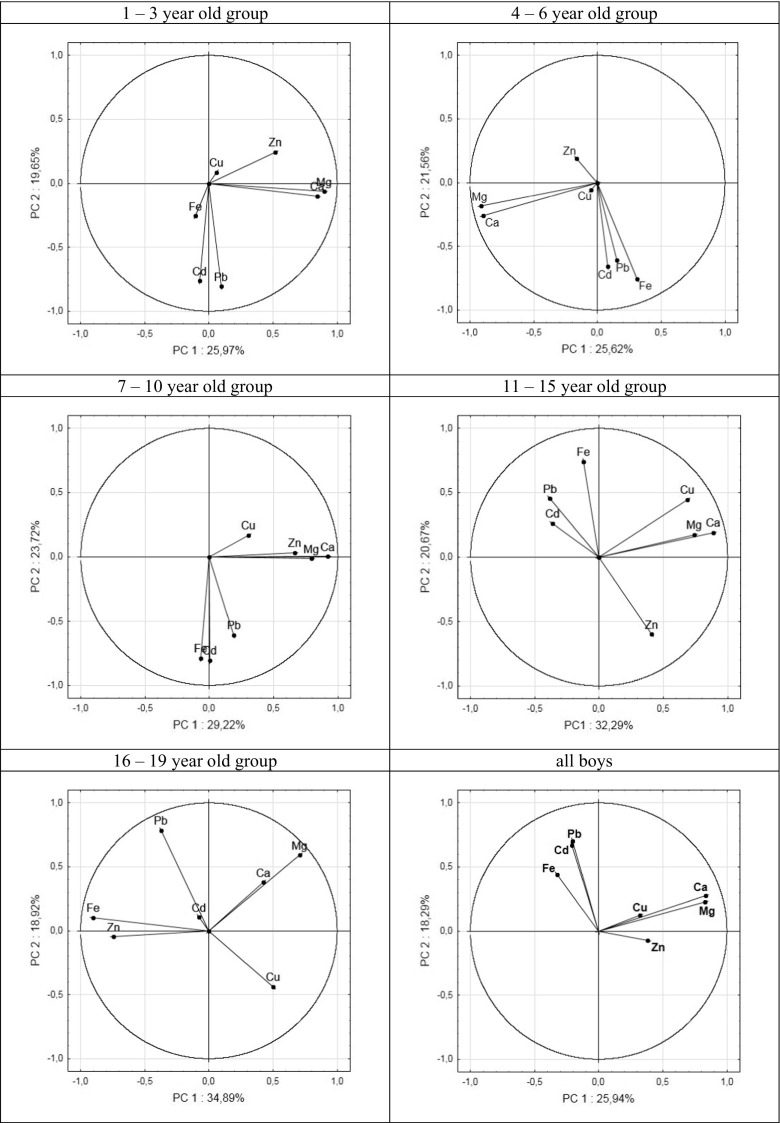
Graphical PCA presentation of relationships between contents of elements in boys’ hair

Additionally, our results were processed using the PCA method. Details of PCA analysis are graphically presented in Figs. 3 and 4. Results shown in the figures should be interpreted in a way that if the individual vectors are closer to each other, positive correlations between the elements represented by the vectors are stronger—and conversely, if the vectors are in opposite direction, the correlation is negative; if they are perpendicular to each other, no correlation occurs.

The analysis of correlation as well the PCA method reveals similar tendencies of correlations between the amount of elements in hair and children age.

Both statistical analyses show congruent trends of correlations between elemental contents in hair. For comparison with the Spearman analysis (Table 4), the strongest positive correlations, resulting from quantitative PCA, were found for the Ca–Mg pair, reaching 0.82 for the 4–6-year-old girl group. The Ca–Mg correlations varied from 0.53 to 0.82 for girls and were always larger than the corresponding Ca–Mg correlations for boys (0.40–0.76), except for the 11–15-year-old group for which the figure was lower (0.53 for girls and 0.61 for boys, respectively). Also, clear positive correlations were observed for the Pb–Cd pair in boys’ hair from all age groups, while only for younger groups of girls (1–3 and 4–6-year-old groups). Other correlations between different elemental pairs were positive in one age group and negative in others. This was observed both for girls and boys. The strongest negative correlations have been found for boys from adolescents (the 16–19-year-old group); for instance, Fe–Mg correlation amounted to −0.56; for Mg–Zn, it was −0.45, for Fe–Cu, −0.34; and Cu–Zn, −0.28. Due to constant value of Cd content in the hair of the 16–19-year-old girl group, there were no correlations found for cadmium and other elements. In general, correlation coefficients calculated by the Spearman test and PCA are similar.

## Discussion

The period of childhood and adolescence is associated with dynamic changes regarding weight and growth gain, in particular for infants, toddlers, and teenagers. These changes are also linked to the gradual development of, among others, digestive, excretory, and endocrine systems. Immature digestive and excretory systems may result in a larger accumulation of heavy metals in the body (see Table 1). Hormonal changes, especially relating to thyroid, parathyroid, pancreas, adrenal cortex, and sexual gland functions which accompany puberty period, also affect the mineral balance in the body. The growth of the child is accompanied by changes in its calorie and food ingredient requirements, metabolic rate, composition of diet (with emphasis on minerals and vitamins), and habits, including food and nutrition customs, which also determine the level of accumulation of elements in the cells of tissues—and also in hair [15].

Similar to content of elements presented in our paper, the relations between the amounts of Ca, Mg, Zn, Fe, Cu, Pb, and Cd in children’s and adolescents’ hair and their age and sex were described by Chojnacka et al. [1], Lech [8], Park et al. [9], and Senofonte et al. [10]. Many factors, both environmental and individual, for example, hair pigmentation [1–3, 16], determine the elemental profile of hair, and we should take them into consideration when interpreting the results of the analysis.

According to the impact of environment on the distribution of elements in children’s hair, correlations were found between contents of elements, including toxic ones, in hair of children of smoking parents, especially in case of Pb, Cd, Fe, Cr, Sb, and Al [17]. Considerably larger Pb amounts have been noted for children living in industrial-complex estates (1.59 μg/g) as compared to downtown areas (0.32 μg/g) [18], and also, 7.16 μg Pb and 0.44 μg Cd per 1 g of hair in comparison to 2.49 μg Pb/g and 0.23 μg Cd/g were measured for “reference group” [19].

Health status of the child is also reflected in the distribution of elements in its hair. Vanaelst et al. [20], who examined contents of selected elements in the hair of girls from the 6–10-year-old group, did not reveal essential correlation between elemental level and consumption of foodstuffs being carriers of these elements. However, they observed a positive relation between hair mineral composition (Ca, Ca/Mg, Ca/P) and individual anthropometric parameters and inverse one between metabo-lic parameters and Ca, Mg, and Ca/P amounts [21]. In the hair of malnourished children, there were considerably lower amounts of Cu, chromium (Cr), and Fe as compared to healthy ones [22]. Other authors [23] tried to evaluate whether hair can be used as a biomarker of marginal zinc deficiency for preschool children. They determined 116 ± 43 μg/g Zn (mean value) in the hair of children from Vancouver. Shah et al. [24] stated lower values of Fe, Zn, Cu, Cr, and nickel (Ni) contents for children with anemia as compared to non-anemic ones. By contrast, significantly higher amounts of Pb and Cd were found in all biological species tested, i.e., blood, urine, and hair of anemic children. These differences were observed both for 1–5 and 6–10-year-old girls and boys. In hair of children with autism, essentially larger values of, among others, Pb, aluminum (Al), Cd, silicon (Si), Cr, Ni, Zn, and Fe and lower for Ca and Cu were determined by Al-Farsi et al. [25]. On the other hand, there was neither a clear upward nor downward trend for elements such as Al, arsenic (As), Ca, Cu, Fe, Pb, Mg, mercury (Hg), and Zn in hair of children with various degrees of mental retardation [26]. Furthermore, significantly lower levels of Fe, Zn, and Mg in the hair of children with growth retardation when compared to the control group were reported by Ozmen et al. [27]. Lech [8] described differences in the amount of elements and their ratios in hair of children and young people with neurological diseases. She found, among others, a lower amount of Mg (20.6 vs. 29.4 μg/g—median values) and a higher quantity of Pb (2.8 vs. 1.6 μg/g—median values) in the hair of children suffering from neuro-logical diseases.

Ranges of reference values worked out for elemental contents in hair are helpful in interpreting the obtained results. Authors [9, 10] present various ranges of these values, particularly for elements such as Ca (120–365 μg/g), Mg (6–24 μg/g), Zn (30–130 μg/g), Fe (7–21 μg/g), and Cu (8–36 μg/g) and heavy metals—Pb (<3 μg/g) and Cd (<0.01–0.20 μg/g). Llorente Ballesteros et al. [28] elaborated reference intervals for amount of 18 elements in hair of 0- to 18-year-old children. Lowest deviations in reported results were observed for Zn, Fe, and Cu. These differences result not only from diversified chemical composition of hair, but also from various analytical and statistical procedures applied in the research.

Examined and established are also quantitative proportions of essential elements and toxic metals, for instance Ca/Mg, Zn/Cu, Ca/Pb, Mg/Pb, Zn/Cd, and Fe/Pb [8, 21, 24, 29, 30]. The quantitative proportions and designated correlations between the content of elements in the study may arise from mutual synergic or antagonistic interactions between them during the absorption process, transmembrane transport, accumulation, and excretion phases. Similar physicochemical properties of the elements and similar size of atoms (for example, Ca and Mg) as well as competition for binding sites in biomolecules are primarily at the root of the interactions between them [31, 32]. In case of Pb and Cd, the cause of strong and significant quantitative correlations may be a common environmental origin of both elements [19]. Inter-element interactions in human hair were also found by other authors. Significant correlations were found especially in the case of Ca and Mg [33, 34] and for a pair of Pb–Cd [19, 28].

The main conclusions of our research are as follows:

- Differences in elements’ contents were found to be dependent on sex and age of children.
- More heavy metals were measured for boys.
- The strongest correlations are observed for Ca–Mg, Ca–Zn, Mg–Zn, and Pb–Cd pairs both for girls and boys.
- The results obtained by both statistical methods show reasonable agreement between correlations found in PCA and Spearman test results. Some differences result from calculation method (Pearson test in case of PCA).

Considering how many factors affect the elemental composition of hair (age, sex, environmental exposure), further research on the content of elements in hair of children and adolescents on a larger population of young people undergoing various environmental influences is very desirable and justified. This should be useful in attempts to create suitable global database for the content of elements in the hair and to evaluate reference values and proper quantitative ratios of essential and toxic elements.
